# Angespannter Arbeitsmarkt — Arbeitskräftemangel nach Corona

**DOI:** 10.1007/s10273-022-3275-4

**Published:** 2022-09-16

**Authors:** ZG Überblick

**Keywords:** J01, J18, J21, J68

## Abstract

Nicht zuletzt durch die Verwerfungen der Coronapandemie ist die Lage derzeit in vielen Branchen angespannt. So sind fehlende Arbeitskräfte vor allem in der Gastronomie, an Flughäfen, in der Pflege und im Handwerk ein Problem. In Summe gab es im ersten Quartal 2022 insgesamt 1,7 Mio. offene Stellen, ein Höchstwert. Ursächlich ist dafür zum einen die Situation, dass sich viele Beschäftigte aufgrund von unsicheren Arbeitsverhältnissen während der Pandemie in andere Branchen umorientiert haben. Das gilt auch für Jobs in der Pflege, die besonderen Belastungen ausgesetzt waren und sind. Eine weitere Ursache ist die Demografie. Es kommen weniger junge Arbeitskräfte nach und die Arbeitsmigration kann nur einen Teil dieser Lücke schließen. Es stellt sich die Frage, wie der Staat auf diesen Arbeitskräftemangel reagieren kann und in welcher Frist die entsprechenden Maßnahmen greifen können.

## Abstract (en)

Due in part to the distortions of the coronavirus pandemic, the current situation in the labour market is tense. The lack of workers is a problem especially in the catering industry, at airports, in nursing and in skilled trades. In total, there were 1.7 million vacancies in the first quarter of 2022, an all-time high. The reason for this is two-fold: on the one hand, many employees have reoriented themselves to other sectors due to insecure employment during the pandemic. This also applies to jobs in nursing, which were and are subject to particular stress. Another cause is demography. Fewer young workers are joining the workforce and labour migration can only fill part of this gap. The question for the government is how to respond to this labour shortage and how long will it take the corresponding measures to take effect.

